# Galectins in Chagas Disease: A Missing Link Between *Trypanosoma cruzi* Infection, Inflammation, and Tissue Damage

**DOI:** 10.3389/fmicb.2021.794765

**Published:** 2022-01-03

**Authors:** Carolina V. Poncini, Alejandro F. Benatar, Karina A. Gomez, Gabriel A. Rabinovich

**Affiliations:** ^1^Laboratorio de Inmunología Celular e Inmunopatología de Infecciones, Instituto de Investigaciones en Microbiología y Parasitología Medica, Universidad de Buenos Aires-Consejo Nacional de Investigaciones Científicas y Técnicas, Buenos Aires, Argentina; ^2^Departamento de Microbiología, Parasitología e Inmunología, Facultad de Medicina, Universidad de Buenos Aires, Buenos Aires, Argentina; ^3^Servicio de Citometría de Flujo, Instituto de Medicina Experimental (IMEX), Academia Nacional de Medicina, Consejo Nacional de Investigaciones Científicas y Técnicas (CONICET), Buenos Aires, Argentina; ^4^Laboratorio de Biología e Inmunología de las Infecciones por Tripanosomátidos, Instituto de Investigaciones en Ingeniería Genética y Biología Molecular, Consejo Nacional de Investigaciones Científicas y Técnicas, Buenos Aires, Argentina; ^5^Laboratorio de Glicomedicina, Instituto de Biología y Medicina Experimental, Consejo Nacional de Investigaciones Científicas y Técnicas, Buenos Aires, Argentina; ^6^Facultad de Ciencias Exactas y Naturales, Universidad de Buenos Aires, Buenos Aires, Argentina

**Keywords:** galectin, *Trypanosoma cruzi*, galectin-1, galectin-3, Chagas disease

## Abstract

*Trypanosoma cruzi*, the protozoan parasite causative agent of Chagas disease, affects about seven million people worldwide, representing a major global public health concern with relevant socioeconomic consequences, particularly in developing countries. In this review, we discuss the multiple roles of galectins, a family of β-galactoside-binding proteins, in modulating both *T. cruzi* infection and immunoregulation. Specifically, we focus on galectin-driven circuits that link parasite invasion and inflammation and reprogram innate and adaptive immune responses. Understanding the dynamics of galectins and their β-galactoside-specific ligands during the pathogenesis of *T. cruzi* infection and elucidating their roles in immunoregulation, inflammation, and tissue damage offer new rational opportunities for treating this devastating neglected disease.

## Introduction

Chagas disease is a major neglected disease in Latin America, affecting around seven million people worldwide and causing 50,000 deaths per year (WHO, 2014; Lidani et al., 2019). It is an anthropozoonosis affecting humans for more than 4,000 years (Guhl et al., 1999; Aufderheide et al., 2004). Although, in the past, the disease was mainly circumscribed to the American continent, there are an increasing number of cases in non-endemic countries mostly due to the migration of infected people from endemic areas (Schmunis, 2007; Lidani et al., 2019). The infection takes place either by vector-borne, congenital routes, blood-borne, and oral or organ-derived transmission (Bern et al., 2019). Successful strategies used to eliminate vectors in some endemic regions, as well as the exhaustive screening in blood banks, highlight the relevance of congenital mother-to-child transmission as the main actor of Chagas disease’s urbanization (Schmunis and Cruz, 2005; Coura and Dias, 2009; De Rissio et al., 2010; Bisio et al., 2011).

The flagellated protozoan *Trypanosoma cruzi* is the etiologic agent of Chagas disease. Glycoproteins and glycolipids play an important role in most of the steps of the complex life cycle of this microorganism, which involves interactions with mammalian hosts and insect vectors from the *Triatominae* subfamily (*Hemiptera*, *Reduviidae*), usually called vinchuca, kissing bug, barbeiro, among others (Tyler and Engman, 2001; De Souza et al., 2010; Garcia et al., 2010). *T. cruzi* has an incredible adaptation capacity that allows infection of more than one hundred mammalian species as well as a great versatility to transmit disease to sylvatic and domiciliary adapted Triatomine vectors (Noireau et al., 2009; Jansen et al., 2020). This flourished life cycle is sharpened by different lineages of *T. cruzi*. Hence, a committee of experts, in 2009, came to the decision to cluster the parasite strains into six discrete typing units (DTU), named TcI to TcVI, and a seventh DTU named TcBat (Zingales et al., 2009), based on biological, biochemical, and genetic diversities. Each DTU exhibits a typical geographic distribution, as well as different predominance in the sylvatic or domestic cycle, variations in their reservoirs, and vectors (Zingales et al., 2012). Until now, it has not been possible to determine a correlation between clinical manifestations and the circulating DTUs in human pathology (Del Puerto et al., 2010; Zingales et al., 2012; Jansen et al., 2020). Thus, despite the usefulness of DTU partition for genetic purposes, the species display a high diversity even in strains present within the same DTU (Roman et al., 2018).

Along its life cycle, *T. cruzi* undergoes biologic, structural, and metabolic transformations to adapt and survive in the different evolving environments. Different forms or parasite stages are epimastigotes and metacyclic trypomastigotes in the vector and amastigotes and blood trypomastigotes in the mammalian host (De Souza et al., 2010; Garcia et al., 2010). Variation in surface mucin glycoconjugates has been described not only in each parasite stage but also in each lineage of the different DTUs (Giorgi and de Lederkremer, 2020). Epimastigotes are rich in mucins that protect them from the action of agglutinins and proteases in the digestive tract (Buscaglia et al., 2006; Villalta et al., 2008). The attachment to peri-microvillar membranes through the interaction of parasite glycoinositolphospholipids with insect-derived glycoconjugates triggers metacyclogenesis and transforms replicative epimastigotes in highly infective metacyclic trypomastigotes that are released by feces and urine during vector feeding (De Souza et al., 2010; Garcia et al., 2010). These forms cannot pass through intact skin but can enter the bloodstream through mucosal tissue or at the biting site after a scratch (Giddings et al., 2010). Once inside the mammalian host, trypomastigotes infect macrophages, fibroblasts, adipocytes, and other cell types, before they reach skeletal, smooth, and cardiac muscle (Landskroner-Eiger et al., 2005; Epting et al., 2010; Ferreira et al., 2011). Invasion is a complex process involving many glycoproteins expressed in metacyclic trypomastigotes, such as gp90, gp82, gp30 y gp35/50 that are differentially expressed in the parasite strains and modulate diverse signaling pathways, which determine efficient internalization of the parasite (Ferreira et al., 2006; Yoshida, 2006; Alves and Colli, 2007; Villalta et al., 2008; Calvet et al., 2012; Romano et al., 2012; Ferri and Edreira, 2021). Other relevant molecules that are implicated in adhesion and invasion processes are mucins, cruzipain, and *trans*-sialidase (TS), a unique protein that reversely transfers sialic acid to β-Gal residues on acceptor molecules present in parasite’s or host’s cell membranes (Vandekerckhove et al., 1992; Buscaglia et al., 2006; De Souza et al., 2010; Calvet et al., 2012; Bartholomeu et al., 2014). Once inside the cell and independently of the route of entry, the parasite transiently persists into the parasitophorous vacuole (PV), where TS has an important role in the protection and maturation of trypomastigotes. Notably, sialic acid transfer activity of TS avoids parasite membranes degradation, and after differentiation, the parasite escapes from the vacuole to the cytosol using TS and other virulence factors (De Souza et al., 2010; Epting et al., 2010). After several rounds of replication, amastigotes start trypomastigote transformation (De Souza, 2002; Waghabi et al., 2005; Alves and Colli, 2007). Finally, and by a poorly understood mechanism, parasites can lyse the cell gaining access to the extracellular space, infect the neighboring cells, or reach the bloodstream, where the cycle restarts (Barrias et al., 2013).

Infection can be divided into two phases: the acute phase mostly presents symptoms that are difficult to ascribe to Chagas disease in a general clinical examination (Pinto et al., 2008); the only exception is the cutaneous damage caused at the site of inoculation, when it occurs (WHO, 2007; Tanowitz et al., 2009; Hemmige et al., 2012). Some people, especially children, may develop life-threatening alterations in the heart and brain during this phase. This number could be as high as 2–5% of the cases in which acute phase is detected (Pinto et al., 2008; Tanowitz et al., 2009; Hemmige et al., 2012; Healy et al., 2015). After 2–4 months, and although the immune system manages to partially control the infection, the chronic asymptomatic stage ensues. It can last throughout the life of the infected individual; the only clinical manifestation could be a subtle degree of myocardial abnormalities in stress echocardiography and Dopler tests that sometimes may lead to sudden death (Punukollu et al., 2007; Tanowitz et al., 2009). However, approximately 30–40% of infected people show clinical alterations, affecting cardiac tissue with an incidence of 20–30%, the digestive organs such as megaesophagus or megacolon with a frequency of 6–10%, or mixed form (Dutra et al., 2009; Hemmige et al., 2012; Viotti et al., 2014). Chronic chagasic cardiomyopathy (CCC), the most frequent manifestation, is a dilated heart disease with focal or disseminated inflammatory infiltrates, destruction of cardiac muscle, progressive fibrosis, and a high prevalence of conduction abnormalities, sinus node dysfunction, complex ventricular arrhythmias, and apical thrombus (Tanowitz et al., 2009; Esper et al., 2015; Healy et al., 2015).

So far, the events that trigger the transition from the chronic asymptomatic to the symptomatic stage are still unknown, and the paradigm shifted over time now accepting that the direct action of the parasite, as well as the immune response generated by the host, are the main mechanisms responsible for cardiac and digestive pathology (Acevedo et al., 2018). Thus, a strong cellular response with a predominance of CD4^+^ T and CD8^+^ T cells producing interferon (IFN)-γ and tumor necrosis factor (TNF)-α has been demonstrated, not only in the heart but also in the blood (Dutra et al., 2009). However, some mechanisms seem to be independent of the parasite persistence, including the development of an autoimmune process, mainly due to molecular mimicry between parasite and host proteins (Levin et al., 1993; Kaplan et al., 1997; Freedman and Lefkowitz, 2004; Smulski et al., 2006; Labovsky et al., 2007; Medei et al., 2007). The list of cross-reactive antibodies is extensive and is out of the scope of this revision (Acosta and Santos-Buch, 1985; Cunha-Neto et al., 1996; Bilate and Cunha-Neto, 2008; Ribeiro et al., 2009). However, CD4^+^ T cells with the ability to recognize host-self antigens have been detected in cardiac tissue of experimentally infected animals, as well as in patients with CCC (Silva-Barbosa and Savino, 2000; Cunha-Neto et al., 2011). In addition, neurogenic degeneration due to denervation of the heart (dysautonomia) and alterations in microcirculation, which generate the ischemic foci observed in hearts from patients with CCC, contribute to the development of this pathology (Bonney and Engman, 2008; Machado et al., 2012). Although the pathogenesis of CCC is multifactorial, it is clear that the presence of different lineages of the parasite, as well as different components of the host’s immune system, may contribute to the progression from an asymptomatic form of the disease toward chronic heart pathology (Viotti et al., 2014; Healy et al., 2015; Acevedo et al., 2018).

## Galectins

Galectins are a family of glycan-binding proteins characterized by the presence of at least one carbohydrate recognition domain (CRD) and a common structural fold. They mainly recognize glycoconjugates containing repetitive structures of the disaccharide *N*-acetyl-lactosamine (Galβ1-4GlcNac or LacNac) (Rabinovich et al., 2007, Van Kooyk and Rabinovich, 2008; Rabinovich and Toscano, 2009; Vasta, 2009; Davicino et al., 2011). Although these proteins bind to the same functional group, their carbohydrate specificity and plasticity in the CRDs are different, which in turn confers diverse functional properties (Vasta, 2012). In fact, each galectin recognizes a distinct set of glycosylated proteins or lipids at the cellular surface, extracellular matrix (ECM), or inside the cell (Wiersma et al., 2013). In general, galectin binding to a single ligand has a low affinity, but their multivalence and the complexity of the glycosylated ligands present on cell glycoproteins turn this binding into reversible high-affinity interactions (Thiemann and Baum, 2016). Terminal modifications such as sialylation, sulfation, or fucosylation on galactose affect galectin binding affinities (Hirabayashi et al., 2002; Rabinovich and Toscano, 2009).

There are at least 15 galectins in mammals expressed in different cells and tissues, including 10 galectins in humans, which are classified in prototype, chimera type, and tandem-repeat type. Prototype galectins (galectins-1, -2, -5, -7, -10, -11, -13, -14, and -15) have one CRD per subunit and are able to form non-covalent dimers; the chimera type galectin-3 has an *N*-terminal region responsible for their oligomerization, whereas tandem-repeat galectins (galectins-4, 6, 8, 9, and 12) have two homolog CRD in the same polypeptide chain, separated by a linker peptide of up to 70 amino acids long. Most of them are secreted through a non-classical mechanism (Van Kooyk and Rabinovich, 2008; Vasta, 2012; Schnaar, 2015), which still remains uncertain. In this regard, recent studies revealed that, in response to stress or infection, galectins are secreted through mechanisms involving non-canonical inflammasome activation and pyroptosis (Russo et al., 2021).

Some galectins, such as galectins-1 and 3, are ubiquitously expressed, whereas others present a more restricted distribution (Van Kooyk and Rabinovich, 2008). By interacting with specific glycoconjugates, galectins can trigger different signaling pathways leading to modulation of several cell processes, including activation, differentiation, apoptosis, receptor turnover, and trafficking (Rabinovich et al., 2007, Van Kooyk and Rabinovich, 2008; Ilarregui et al., 2009; Rabinovich and Toscano, 2009). By tempering these processes, galectins can control immune homeostasis, either in normal or pathologic conditions, with beneficial or detrimental effects to host tissues (Rabinovich and Croci, 2012). Moreover, galectins can modulate host–pathogen interactions and serve as mediators of immune evasion mechanisms (Davicino et al., 2011).

Interestingly, galectins have been proposed to bind glycan moieties present on the surface of viruses, fungi, bacteria, and parasites (Vasta, 2009), highlighting the role of these lectins as pattern recognition receptors. Under certain conditions, they can directly interact with receptors in host cells and inhibit the interaction of pathogens or even cross-link and immobilize them at the ECM, ultimately blocking infection (reviewed by Vasta, 2020). Furthermore, galectins are abundant both in the intracellular and the extracellular compartments and can influence infection, dissemination, or pathogen eradication through different mechanisms. Through direct or indirect pathways, pathogens themselves can up- or downregulate expression, concentration, and subcellular distribution of galectins at sites of infection (Levroney et al., 2005; Lujan et al., 2018). Strikingly, galectins may indirectly control pathogen persistence or elimination by positively or negatively shaping antimicrobial immunity (Sato et al., 2014; Nita-Lazar et al., 2015; Davicino et al., 2017; Xue et al., 2017).

## Galectins and *Trypanosoma cruzi*

**FIGURE 1:**
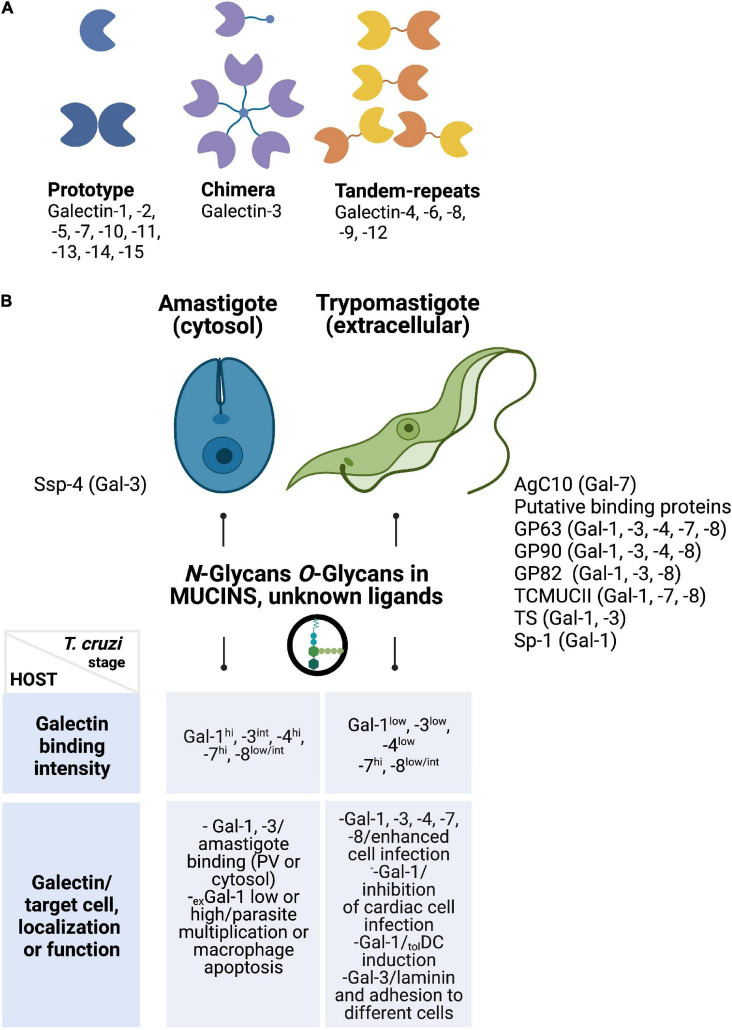
**(A)** Structural classification of galectins. Some members of the prototype (galectin-1 and -7), chimera-type (galectin-3), and tandem-repeat type (galectins-8 and -9) galectins have been associated with *T. cruzi* recognition. **(B)** Galectins preferentially recognize parasite stages found in the mammalian host (amastigotes and trypomastigotes). Only galectin-7 recognizes epimastigotes (not shown). Potential receptors that bind human galectins (upper panel) and the intensity of some of these interactions reported by Pineda et al. (2015a) and others are summarized here (lower panel). Target cells, cellular localization, and/or properties of endogenous galectins during *T. cruzi* infection are enumerated in the lower panel. Ssp-4, stage-specific protein 4; AgC10, surface mucin AgC10; GP63, surface protease 63; GP82-90, surface glycoprotein (82–90); TcMUCII, *T. cruzi* mucin II; TS, *trans*-sialidase; Sp-1, surface protein 1.

Previous studies described differential binding of human galectins to *T. cruzi*, demonstrating selective recognition of different parasite stages by these glycan-binding proteins. Interestingly, they can display a particular binding profile related to the parasite’s genetic background (Pineda et al., 2015a). *T. cruzi* is highly glycosylated, and surface glycoconjugates differ among biological stages of the parasite (de Lederkremer and Agusti, 2009). As described earlier, the parasite surface is enriched in mucins, which are complex glycoproteins displaying a dense array of *O*-linked oligosaccharides that constitute a coat that protects the parasite from the host and mediates interactions with host receptors and glycan-binding proteins. In general, most of the components are mucins-like proteins anchored to the parasite surface by glycosylphosphatidylinositol. Of note, carbohydrates are the major components of these molecules and account for up to 60% of their molecular mass (Buscaglia et al., 2006). Here, we review several interactions that take place between galectins and *T. cruzi* that control parasite invasion and immunity (Figure 1).

## Galectin-1 and *Trypanosoma cruzi* Infection

Galectin-1, a prototype member of the galectin family, is highly expressed by different cell types, including immune cells, epithelial cells, endothelial cells, and adipocytes, at sites of infection and inflammation (Toscano et al., 2018). The binding affinity of galectin-1 for LacNAc-expressing glycoconjugates depends on the structure and nature of the glycoconjugate, presented either as a glycoprotein or glycolipid, and on the galactose linkage type and the branches on complex *N*-glycans. In general, galectin-1 binding to a single ligand is a low-affinity interaction, but the complexity of the glycan ligands present on cellular glycoproteins can turn this association into a reversely high-affinity interaction (Thiemann and Baum, 2016). Of note, this lectin has a high affinity for complex *N*-glycans, and the binding to LacNac is interrupted by α2,6-sialylation on the terminal galactose. In addition, elongated core 2-*O*-glycans, glycosphingolipids, and some gangliosides have been described to bind galectin-1 (Rabinovich and Toscano, 2009). The binding to multivalent glycans on the cell surface promotes galectin-1–glycan interactions (Bourne et al., 1994). This effect favors proximity between two or more cells, adhesion of cells to glycosylated surfaces, and the formation of lattices at the cell surface, which facilitates receptor clustering, signaling, turnover, and endocytosis (Kutzner et al., 2020). Moreover, these effects rely on tissue-specific expression, distribution, and local concentrations of this lectin (Vasta, 2020). In general, galectin-1 displays anti-inflammatory and pro-resolving capacity by targeting multiple immune cells, including lymphoid and myeloid cells. In fact, this lectin influences cellular activation, differentiation, and survival of T cells, B cells, and dendritic cells (DCs) (Sundblad et al., 2017). By virtue of these mechanisms, this lectin has been shown to foster cancer immunosuppression (Rabinovich and Conejo-García, 2016), promote resolution of autoimmune disorders (Rabinovich et al., 1999; Toscano et al., 2006, 2018), and dampen immunity against several pathogens (Davicino et al., 2011; Vasta, 2020). Interestingly, galectin-1 has been proposed to serve as a danger-associated molecular pattern secreted in response to infection inflammation and stress (Sato et al., 2009; Vasta, 2020; Russo et al., 2021).

An early report in Chagas disease demonstrated the upregulation of galectin-1 in cardiac tissues of CCC patients and revealed an increased titer of circulating anti-galectin-1 autoantibodies in sera from these individuals (Giordanengo et al., 2001). More recently, galectin-1 was found to be upregulated in the myenteric plexus ganglia of patients with Chagas disease, suggesting a possible association between this lectin and the ganglionitis in the chagasic megacolon (Beghini et al., 2017). Interestingly, the presence of anti-galectin-1 antibodies has also been documented in patients with autoimmune neurological disorders (Lutomski et al., 1997), systemic lupus erythematosus (Montiel et al., 2010), and rheumatoid arthritis (RA, Xibillé-Friedmann et al., 2013). Of note, not only auto- anti-galectin-1 antibodies but also changes in galectin-1 serum levels have been found in these autoimmune disorders (Montiel et al., 2010; Mendez-Huergo et al., 2018). Likewise, elevated levels of galectin-1 have been detected in sera from *T. cruzi-*infected patients in both the asymptomatic and cardiac phases of this inflammatory disease (Benatar et al., 2015).

Chronic chagasic disease is multifactorial cardiomyopathy, and the mechanisms involved in the progression to severe manifestations have not been fully elucidated. It is well accepted that both the persistence of the parasite and immune effector cells trigger tissue damage (Marin-Neto et al., 2007). An inflammatory microenvironment generated by the infection modifies the expression of metalloproteinases, galectins, and cytokines, including transforming growth factor-β, which contribute to the development of myocarditis, tissue remodeling, and fibrosis, thus influencing the progression of the parasite cycle and stimulation of cardiac tissue alteration (da Costa et al., 2019). As observed in patients with severe CCC (Giordanengo et al., 2001), Seropian et al. (2013) described the upregulation of galectin-1 in human cardiac tissue from patients with end-stage chronic failure (Seropian et al., 2013). Interestingly, galectin-1 was overexpressed in cardiac cells exposed to pro-inflammatory cytokines or hypoxic stimuli. Mice lacking galectin-1 (*Lgals1^–/–^)* presented exacerbated symptoms with more inflammatory cells and fewer regulatory T cells (Tregs) in hearts compared with their wild-type (WT) counterparts (Seropian et al., 2013), suggesting a protective role for this lectin in cardiac tissue homeostasis.

In the early 1980s, Henriquez et al. (1981) documented *in vitro* the effect of pretreatment of three distinct cell types (Vero, MA-103, and chick muscle cells) with different lectins to prevent *T. cruzi* infection. More recently, galectin-1 has been shown to inhibit the infection of cardiac cells exposed to *T. cruzi* in culture, using two strains of the parasite with different genetic backgrounds. Interestingly, a parasite strain-dependent glycophenoytpe of cardiac cells was observed, characterized by a reduction in galectin-1-specific ligands on the surface of cells infected with the most virulent strain (Tulahuen, Tul). Protection against *T. cruzi* infection mediated by galectin-1 was confirmed in the experimental *T. cruzi* infection model, where *Lgals1^–/–^* animals infected with Tul strain displayed enhanced mortality and parasite load in cardiac tissue compared with WT mice. Thus, modulation of galectin-1–glycan interactions in cardiac cells may influence parasite-driven heart injury (Benatar et al., 2015).

In the past years, an increasing number of reports documented the role of galectin-1 within innate and adaptive immune compartments (Toscano et al., 2018). Through its ability to induce tolerogenic DCs and Tregs (Ilarregui et al., 2009) and promote apoptosis of activated Th1 and Th17 cells (Toscano et al., 2007), galectin-1 promotes tumor-immune escape (Cagnoni et al., 2021), feto-maternal tolerance (Blois et al., 2007), and resolution of autoimmune neuroinflammation (Ilarregui et al., 2009). Interestingly, *T. cruzi* infection upregulates the expression of galectin-1 in different immune cells, including B cells and macrophages (Zúñiga et al., 2001a,b). Of note, galectin-1 produced by activated B cells triggered apoptosis of activated T cells and reduced IFN-γ production by these cells (Zúñiga et al., 2001a). In addition, J774 macrophages responded to *T. cruzi* trypomastigotes by releasing high amounts of galectin-1 (Zúñiga et al., 2001b). Recombinant galectin-1 enhanced microbicidal activity and controlled survival of *T. cruzi*-infected macrophages. Under low concentrations of this lectin, splenic macrophages from infected mice showed active replication of the parasite, low interleukin (IL)-12 production, and inhibition of nitric oxide production, consistent with alternative M2 macrophage activation (Correa et al., 2003). On the other hand, a high concentration of galectin-1 promoted dose-dependent apoptosis of macrophages and inhibition of parasite replication (Zúñiga et al., 2001b).

Compelling evidence shows that *T. cruzi* can directly influence the function of DCs, interfering with the development of adaptive immune responses. Van Overtvelt et al. (1999) reported that human monocyte-derived DCs could be infected by *T. cruzi*, preventing optimal activation and blunting production of pro-inflammatory cytokines. Subsequently, it was demonstrated that the parasite promotes IL-10-producing bone marrow-derived tolerogenic DCs (Poncini et al., 2008). In this regard, galectin-1 has emerged as a decisive factor instructing DCs to become tolerogenic. *Lgals1^–/–^* mice are refractory to the regulatory effects of *T. cruzi*, preserving immunogenicity of DCs upon parasite stimulation. Intradermal infection with the high virulent RA strain of *T. cruzi* induced early recruitment of DCs to draining lymph nodes and local upregulation of galectin-1. Of note, this lectin was expressed in the spleen during the acute phase of infection. Surprisingly, and in contrast with the results obtained by Benatar et al. (2015) following the intraperitoneal route of infection, *Lgals1^–/–^* mice presented enhanced resistance to acute *T. cruzi* infection and low parasite burden in tissue, with a major susceptibility in female compared with male animals. Enhanced susceptibility to *T. cruzi* infection in WT mice and persistence of the parasite in infected tissues were driven by a regulatory circuit initiated by upregulation of galectin-1, which elicited tolerogenic DCs, Tregs, and inhibition of antigen-specific T cell responses (Poncini et al., 2015). The discrepancies described in the experimental models of infection using *Lgals1^–/–^* mice could be related to distinct parasite administration routes, the presence of different phagocytes at the site of inoculation, and the local immune response triggered by different *T. cruzi* strains (Barbosa et al., 2019). In addition, it was demonstrated that *T. cruzi* could modulate the glycophenotype in some cell types suggesting an exquisite evolutionary condition of this parasite that allows manipulation to persist in host cells. This plasticity would also add complexity to the outcome of the infection that is affected by the parasite strain (Tul, Brazil, or RA), the route of infection (intraperitoneal or intradermoplantar inoculation), and the immune responses triggered by the parasite. In this regard, strain-dependent discrepancies have been previously described in other parasite infection models (Toscano et al., 2012). Thus, galectin-1 emerges as a central component of the infection machinery co-opted by *T. cruzi* to persist in the host, evade immune responses, and promote tissue damage. However, despite considerable progress, the mechanisms leading to upregulation of galectin-1 synthesis by *T. cruzi* and the signaling pathways underlying galectin-1–glycan signaling in response to parasite infection remain largely unexplored.

## Galectin-3 and *Trypanosoma cruzi* Infection

Galectin-3, the chimera type member of the galectin family, has a high affinity to oligosaccharides bearing 2- or 3-*O*-α-substituents on the outer galactose residue of glycans, such as NeuNAcα2,3 lactosamine or the A-blood group structure GalNAcα1,3 [Fucα1,2] Galβ1,4GlcNAc (Barboni et al., 2000; Krzeminski et al., 2010).

Galectin-3 is the best-studied galectin in the context of *T. cruzi* infection. It has been shown to promote *T. cruzi* adhesion and invasion and modulate interactions between the parasite and the host immune system. An early report came from Moody et al. (2000), who showed that *T. cruzi* adhesion to mammalian cells was favored by interactions between parasite mucins and human laminin *via* galectin-3-mediated bridging. *In vitro* experiments showed that human galectin-3 significantly increased the attachment of trypomastigotes to laminin-coated plates but not to collagen. As this association was blocked by lactose in a dose-dependent manner, it has been proposed that interactions between *T. cruzi* mucins and this lectin involve its conserved CRD. Interestingly, the authors proposed the presence of a parasite-derived galectin-3, although genes encoding this protein have not been reported in *T. cruzi* (Turner et al., 2002). Later, the role of human galectin-3 in favoring the adhesion of the infected forms of *T. cruzi* was confirmed using different types of cells, including human coronary artery smooth muscle (CASM) cells, peritoneal macrophages, DCs, cardiac fibroblasts, and Hela cells (Kleshchenko et al., 2004; Vray et al., 2004; Machado et al., 2014; Souza et al., 2017a,b; Chain et al., 2020). By treating CASM cells with galectin-3 antisense oligonucleotides, the attachment of *T. cruzi* diminished dramatically, and this effect was reverted when recombinant galectin-3 was added to the culture media (Kleshchenko et al., 2004; Chain et al., 2020). Exogenous galectin-3, which is secreted by the same cells, could bind glycans present on the surface of both the parasite and human CASM cells in a lectin-like manner. These data indicate that the autocrine action of galectin-3 is essential for parasite attachment and invasion to host cells. However, a similar experimental approach used in previous studies was not successful at demonstrating that adhesion of trypomastigotes to spleen-derived murine DCs line D2SC-1 requires expression of galectin-3. In fact, D2SC-1 cells stably transfected with galectin-3 antisense revealed no differences in the percentages of infected cells or in the number of amastigotes per cell compared with its WT counterpart. The rationale behind this discrepancy was mainly based on the fact that *de novo* synthesis of galectin-3 might occur during the experimental procedure. The authors then moved forward to show that the expression of galectin-3, as well as galectin-3-specific ligands, was upregulated in splenic DCs isolated from BALB/c mice infected with trypomastigotes (Vray et al., 2004). These findings supported the notion that *T. cruzi* infection can modulate lectin expression using both *in vitro* and *in vivo* experimental models and could have a direct effect on the capacity of parasites to migrate and attach to host cells.

Of note, *T. cruzi* also enhanced the expression of other components of the ECM, such as laminin γ-1 and thrombospondin. By taking advantage of bioinformatics approaches and using these proteins together with galectin-3 as seed nodes, Cardenas et al. (2010) constructed an interactome network highlighting how *T. cruzi* could modulate the human ECM to facilitate cellular infection and trigger disease progression during the early phase of the infection (Nde et al., 2012). However, experimental models of chronicity spilled over the idea that galectin-3 was modulated only during the early stages of infection. In fact, galectin-3 was found to be upregulated in macrophages of the inflammatory infiltrate in hearts from C57Bl/6 mice for a total of 8 months post-infection (Soares et al., 2011). The expression of galectin-3 returned to basal levels of naïve mice when chagasic animals were injected with bone marrow cells or were treated with granulocyte colony-stimulating factor (Soares et al., 2011; Vasconcelos et al., 2013). In both cases, reduction of this lectin was associated with a recovery of heart tissue, lower inflammatory responses, and attenuated fibrosis.

Accordingly, *T. cruzi* also induced elevated levels of galectin-3 in the thymus and modulated the functionality of thymocytes. Infection of BALB/c mice with the blood-derived *T. cruzi* parasites of the Colombian strain induced upregulation of this lectin in both the cortical and medullary compartments of the thymus. In addition, galectin-3 was also found to be increased in the cytoplasm of the CD4^+^/CD8^+^ thymocytes and fostered the migration of this cell subset to peripheral lymphoid organs secondary to parasite infection (Silva-Monteiro et al., 2007). Similarly, the expression of galectin-3 was upregulated in *T. cruzi-*infected mesenchymal stromal cells, which are multipotent stem cells with the capacity to differentiate into mesoderm-derived cell lineages, such as chondrocytes, osteocytes, and adipocytes (Souza et al., 2017a). In further studies, it would be interesting to investigate whether galectin-3 helps to “hide” the parasite in tissues where immune responses are not fully active.

Remarkably, the role of galectin-3 during *T. cruzi* infection is not restricted to modulation of cellular attachment, as it also extends to invasion and intracellular trafficking of the replicative form of the parasite in mammals (Machado et al., 2014; Chain et al., 2020). Immunofluorescence staining showed recruitment of galectin-3 at sites of parasite entry using different cell types, i.e., peritoneal macrophages from C57BL/6 mice, mouse embryonic fibroblasts, and breast carcinoma cell lines (SKBR) cells. After 6 h post-infection, galectin-3, but not the lysosomal membrane proteins (LAMP)-2, was found to be accumulated around *T. cruzi* amastigotes, supporting the notion that this lectin encloses the parasite that has recently lysed the phagolysosome and escaped to the cytoplasm (Machado et al., 2014). The recruitment of galectin-3 in the lysed vacuoles was first described in cells infected with Gram-negative and Gram-positive bacteria, forming the so-called galectin-3-containing structures (Paz et al., 2010). In both models of infection, galectin-3-containing structures depend on the galectin-3 CRD, but this interaction seemed not to comprise the microorganism but galactose-containing glycoconjugates present in the membrane of the lysed vacuole (Paz et al., 2010; Machado et al., 2014). Mirroring these data, galectin-3, plus LAMP-1 and actin filaments, was found around the PV containing amastigotes and trypomastigotes in murine peritoneal macrophages at early times of infection (Reignault et al., 2014). However, immunofluorescence assays carried out after 96 h post-infection showed a diminished number of parasites in cells in the absence of galectin-3, which did not interfere with *T. cruzi* escape from PV into the cytoplasm. This finding opened a conundrum with respect to the role of galectin-3 in the process of parasite intracellular trafficking. Moreover, recent studies demonstrated that the stage-specific protein 4, a glycoprotein present in the surface of amastigotes, could be involved in galectin-3 recruitment during host cell invasion (Florentino et al., 2018). Recently, it was reported that *T. cruzi* induces the cleavage of galectin-3 through different parasite proteases, including Zn-metalloproteases and collagenases, rendering a truncated form of the lectin, which retains an intact CRD but impedes its oligomerization. This phenomenon raised the question of possible causes and mechanisms through which *T. cruzi* affects lectin structure. Pineda et al. (2020) demonstrated that parasite death induced by long-term interactions between galectin-3 and *T. cruzi* is avoided by cleavage of the lectin *N*-terminal domain. Through this mechanism, the parasite likely counteracts galectin-3-driven immunity and host microbicidal activity, highlighting a possible strategy developed by parasites to survive inside mammalian hosts (Pineda et al., 2020).

There is ample evidence stressing the role of galectin-3 in immune responses mounted during *T. cruzi* infection and the outcome of cardiac alterations. This lectin was found to be highly expressed in B cells isolated from BALB/c mice infected with *T. cruzi* (Acosta-Rodríguez et al., 2004). This elevated expression was also detected when resting B cells were activated *in vitro* with different stimuli, such as lipopolysaccharide and F(ab’)2 anti-μ and anti-CD40 antibodies, reaching a maximum effect after a long period of incubation. In both cases, galectin-3 was upregulated in the presence of IL-4, a cytokine that favored B cell survival and the generation of a memory phenotype (Rothstein et al., 2000; Acosta Rodriguez et al., 2003). However, IL-4 activity was abolished when the synthesis of endogenous galectin-3 was interrupted, clearly demonstrating the existence of a mechanism of cross-talk between IL-4 and galectin-3 in the context of acute Chagas disease. Thus, endogenous galectin-3 could serve as a possible mechanism used by the parasite to evade B cell responses. In this regard, inhibition of endogenous galectin-3 during acute *T. cruzi* infection reduced parasitemia by promoting plasma cell formation and secretion of immunoglobulin (Ig)M and IgG (Acosta-Rodríguez et al., 2004).

Interestingly, infected *Lgals3^–/–^* mice showed a drop in serum levels of Th1 and Th2 cytokines, including IFN-γ, IL-2, IL-5, IL-6, IL-10, and TNF, compared with WT mice; these differences were significantly pronounced at 14 days but ceased at 28 days post-infection. Also, the expression of IL-5, IFN-γ, and TLR4 was also significantly diminished in splenocytes of galectin-3-deficient mice. These data, which correlated with the increase in parasitemia, demonstrated that galectin-3 is involved in the initial anti-*T. cruzi* response that connects innate and adaptive immunity. In line with these findings, this lectin favored the occurrence of cardiac alterations, as hearts from *Lgals3^–/–^* mice showed fewer inflammatory infiltrates and prominent signs of fibrosis. Of note, this effect was accompanied by increased parasitemia in the absence of parasite load. Delving into antigen-presenting cell functionality, a lower activation state was evidenced by a reduced expression of the co-stimulatory molecule CD80 and decreased IL-1 and TNF-α production after *in vitro* infection with the parasite. As *Tlr1* and *Tlr4* messenger RNA levels were also affected in *Lgals3*^–/–^ DCs, one might speculate that endogenous galectin-3 controls DC responses by interacting with these receptors. Overall, these findings support the notion that galectin-3 is associated with the outcome of heart injury and inflammation in the context of *T. cruzi* infection (Pineda et al., 2015b).

It is important to emphasize that, similar to galectin-1, the parasite strain used is clearly a relevant factor that governs host–parasite interplay and should be clearly specified in all studies performed. Thus, by exploring the development of cardiac alterations in the murine infection and its association with galectin-3 expression using different human isolates of *T. cruzi* fitting with DTUs I, V, and VI, only animals infected with the former strains developed moderate to severe myocarditis (Ferrer et al., 2014). Immunohistochemical analysis revealed that the myocardial fibrotic areas correlated with higher expression of galectin-3, suggesting a role of this lectin as a putative marker of cardiac progression in Chagas disease (Ferrer et al., 2014). In addition, specific binding of galectin-3 changed among the different life stages of the parasite and also among the six lineages analyzed, probably due to dissimilar glycoconjugates decorating *T. cruzi* cell surface (Pineda et al., 2015a).

A widespread study using animal models and human settings underscored the involvement of galectin-3 in the development of heart disease at the chronic stage of Chagas disease. Souza et al. (2017a) confirmed in an experimental murine model that *T. cruzi* induced galectin-3 expression in the heart and favored the recruitment of infiltrating inflammatory cells at day 30 post-infection, whereas signs of fibrosis appeared after 180 days of the prime infection. Interestingly, the expression of galectin-3 was not limited to CD3^+^ T cells but was also verified in macrophages and fibroblasts. In the latter, both exogenous addition of galectin-3 and its silencing demonstrated that this lectin promotes cellular proliferation *via* its CRD. Even more, treatment with *N*-acetyl-D-lactosamine in mice chronically infected with *T. cruzi* did not improve their cardiological performance but significantly reduced inflammatory infiltrates and fibrosis (Souza et al., 2017b). These effects were accompanied by a considerable reduction of pro-inflammatory cytokines, such as TNF and IFN-γ, transcription factors associated with development and regulation of adaptive immune responses including T-bet (Th1), GATA-3 (Th2), and FoxP3F (Tregs), and modulation of chemokines including chemokine ligand 8 (CCL8) and the chemokine receptor 5 (CCR5), compared with untreated infected animals. However, *IL-10* messenger RNA levels were not altered when compared with naïve mice. Explants of heart from chronic cardiac patients obtained during transplantation also extended previous observation regarding galectin-3 expression in the inflamed areas of this tissue. This study demonstrated the critical roles of galectin-3 in the progression of Chagas cardiac pathology, highlighting its potential role as a therapeutic target in the management of the disease. In this sense, the use of compounds such as 1,2,3-triazole arylsulfonamide-derived-3-*O*-galactosides, which inhibit galectin-3, diminished *T. cruzi* invasion of LLCMK2 cells, a cell line derived from monkey kidney epithelial cells (Marchiori et al., 2017).

Studies in WT and galectin-3 knockout Swiss mice during the acute infection with *T. cruzi* Y strain contributed to unveiling the effect of galectin-3 in the modulation of serum cytokines. Although IFN-γ and TNF were elevated in the *Lgals-3^–/–^* animals, upregulation of IL-4, IL-6, IL-10, and IL-17 were also evident at 15 days post-infection, inducing a shift toward Th17 and Th2 responses that could be explained by mechanisms involving galectin-3 modulation of the innate immune response during *T. cruzi* primo-infection (Chain et al., 2020). Noteworthy, the lack of galectin-3 led to a drop in systemic parasitemia and increased mouse survival. This study suggested that murine models of infection involved a higher parasitic Y load when compared with those used by Pineda et al. (2015a), whereas da Silva et al. (2017) used another *T. cruzi* strain. Furthermore, this investigation revealed the role of galectin-3 in cell survival by demonstrating that parasite-infected peritoneal macrophages or Hela cells required this lectin to escape apoptotic cell death. Infected galectin-3-depleted cells showed not only loss of mitochondrial membrane potential but also an increase in caspase-3 activity and elevated proteolytic processing of poly (ADP-ribose) polymerase after 4 and 8 h post-infection (Pineda et al., 2015b; da Silva et al., 2017; Chain et al., 2020). In line with these findings, chronically infected *Parp1^–/–^* mice showed lower levels of galectin-3, along with additional markers of fibrosis such as transforming growth factor-β and vimentin in heart-resident CD68^+^ macrophages, compared with their WT counterparts (Choudhuri and Garg, 2020). Hence, a signaling pathway connecting galectin-3 with poly (ADP-ribose) polymerase seems to be involved in the apoptotic and fibrotic processes occurring in cardiac tissue during *T. cruzi* infection. Finally, da Silva et al. (2017) also showed, using an infection model of the *T. cruzi* CL strain, that the absence of galectin-3 increased the replication of intracellular parasites in mouse peritoneal macrophages and cell lysis while augmenting blood parasite levels and reducing mast cell recruitment to the heart (da Silva et al., 2017). The discrepancies found among different reports emphasized the critical relevance of parasite strain and doses, inoculation routes, and acute *versus* chronic murine models of *T. cruzi* infection.

When the impact of galectin-3 on digestive manifestations of chronic *T. cruzi* infection was studied, it was observed that the myenteric plexus ganglia in biopsied fragments of the colon from patients with megacolon presented higher expression of galectin-3 along with galectins-1 and 9 (Beghini et al., 2017). A recent study confirmed this observation revealing an increased number of cells expressing galectin-3 that are associated with major staining of collagen type I and type III in tissue areas, suggesting the occurrence of ganglionitis and myositis, two pathological traits implicated in this process. Although galectin-3 was mainly upregulated in the group of patients presenting megacolon with intact intestinal mucosa but not in those with an ulcerated intestinal mucosa and/or mucosal hypertrophy, it has been proposed that this lectin could be a key factor implicated in the progression of colon pathology in the context of Chagas disease (Garvil et al., 2020). Thus, galectin-3 controls not only parasite infection and immune responses but also cardiac and digestive pathology.

## Other Galectins Implicated in *Trypanosoma cruzi* Infection

Despite significant evidence demonstrating the role of galectins-1 and 3 during *T. cruzi* infection, the role of other members of the galectin family in the context of Chagas disease is still uncertain.

In a comprehensive study focused on the interaction between human galectins and the three forms belonging to the six DTUs of *T. cruzi*, Pineda et al. (2015b) showed, using the Y strain, that galectins-7 and 8 bound mainly to trypomastigotes, whereas galectins-1 and 4 presented higher affinity for amastigotes. Interestingly, only galectin-7 showed the binding capacity to epimastigotes, the non-infective *T. cruzi* stage (Pineda et al., 2015b). Moreover, these differences were not only observed among the genetic lineages of the parasite but were also evident among the diverse strains within them. Galectin binding to *T. cruzi* was disrupted in the presence of lactose, highlighting the relevance of the CRD in galectin–parasite interaction. These findings demonstrated that the glycan profile exposed on the parasite surface varies during its life cycle, suggesting that it could be one of the mechanisms used by the parasite to survive in invertebrate and vertebrate hosts. Like galectin-3, the majority of tested galectins, mainly galectin-8, fostered adhesion of trypomastigotes to different cells lines, including THP-1 (human monocytic cells), LLC-MK2 (rhesus monkey kidney epithelial cells), CaCo (human colorectal adenocarcinoma cells), and HL-1 (cardiac myocyte cells). Particularly, parasite adhesion induced by galectin-7 was enhanced with higher concentrations of this lectin, suggesting the need for homodimer formation for this effect. These results emphasize the distinct roles of different galectins during *T. cruzi* infection. Thus, galectin-7, which is mostly expressed in stratified epithelia (Advedissian et al., 2017), could be one of the first mediators that favor the entry of the parasite to host cells.

Galectin-8 has recently been studied in WT and C57BL/6J *Lgals8^–/–^* mice chronically infected with Ac strain, belonging to DTU TcI (Bertelli et al., 2020). Lack of galectin-8 induced higher inflammatory infiltrates, mainly neutrophils and macrophages in heart tissue, and IFN-γ production, but systemic parasitemia and survival rate remained similar. Of note, galectin-8 was increased in the heart of infected WT mice (Bertelli et al., 2020). These findings highlight the anti-inflammatory role of galectin-8 in chronic *T. cruzi* infection.

Finally, galectin-9 was found to be upregulated in biopsy fragments of the colon from patients with chronic Chagas disease presenting megacolon (Beghini et al., 2017).

Thus, despite a major role for galectins-1- and 3 in Chagas disease, galectins-7, 8, and 9 have also been shown to control anti-parasite immunity and modulate tissue damage.

## Conclusion and Future Directions

Galectins play decisive roles during the life cycle of *T. cruzi*. Whereas most studies have focused on the role of galectins-1 and 3 in parasite adhesion, invasion, immune evasion, and tissue damage, other galectins, including galectins-7, 8, and 9, also play relevant functions in the context of Chagas disease. Future studies should be aimed at examining, in parallel using the same parasite lineages and strains, the unique and distinctive roles of different members of the galectin family in mice lacking galectins in relevant tissues. Moreover, given the preferential recognition of individual members of the galectin family for different glycans, further work using glycan array technologies (Arthur et al., 2014) should be performed to dissect biochemical determinants of *T. cruzi*–galectin interactions. Furthermore, the prognostic value of soluble galectins and anti-galectin autoantibodies in sera from patients with Chagas disease should be confirmed in a larger cohort of patients. Finally, given the design of selective galectin inhibitors (Cagnoni et al., 2016), the therapeutic activity of these antagonists should be evaluated in preclinical models of Chagas disease and further validated in *in vitro* settings of infection of human cells with the ultimate goal of finding new treatments for this neglected disabling disease that affects more than two to three million people worldwide.

## Author Contributions

CP, AB, and KG contributed to the selection of the manuscript, integration of studies, and writing of the original draft. GR contributed to the conceptualization and integration of the review. All authors approved the submitted version.

## Conflict of Interest

The authors declare that the research was conducted in the absence of any commercial or financial relationships that could be construed as a potential conflict of interest. The handling editor declared a past co-authorship with one of the authors GR.

## Publisher’s Note

All claims expressed in this article are solely those of the authors and do not necessarily represent those of their affiliated organizations, or those of the publisher, the editors and the reviewers. Any product that may be evaluated in this article, or claim that may be made by its manufacturer, is not guaranteed or endorsed by the publisher.
